# Diagnostic Value of Multiple Tumor Markers for Patients with Esophageal Carcinoma

**DOI:** 10.1371/journal.pone.0116951

**Published:** 2015-02-18

**Authors:** Jun Zhang, Zhenli Zhu, Yan Liu, Xueyuan Jin, Zhiwei Xu, Qiuyan Yu, Ke Li

**Affiliations:** 1 Department of Preventive Medicine, Shantou University Medical College, No. 22 Xinling Road, Shantou, Guangdong, 515041, China; 2 Department of International Center for Liver Disease Treatment, 302 PLA hospital, No. 100 Xisihuan Road, Beijing, 100017, China; 3 Department of Medical Quality Control, 302 PLA Hospital, No. 100 Xisihuan Road, Beijing, 100017, China; University of North Carolina School of Medicine, UNITED STATES

## Abstract

**Background:**

Various studies assessing the diagnostic value of serum tumor markers in patients with esophageal cancer remain controversial. This study aims to comprehensively and quantitatively summarize the potential diagnostic value of 5 serum tumour markers in esophageal cancer.

**Methods:**

We systematically searched PubMed, Embase, Chinese National Knowledge Infrastructure (CNKI) and Chinese Biomedical Database (CBM), through February 28, 2013, without language restriction. Studies were assessed for quality using QUADAS (quality assessment of studies of diagnostic accuracy). The positive likelihood ratio (PLR) and negative likelihood ratio (NLR) were pooled separately and compared with overall accuracy measures using diagnostic odds ratios (DORs) and symmetric summary receiver operating characteristic (SROC) curves.

**Results:**

Of 4391 studies initially identified, 44 eligible studies including five tumor markers met the inclusion criteria for the meta-analysis, while meta-analysis could not be conducted for 12 other tumor markers. Approximately 79.55% (35/44) of the included studies were of relatively high quality (QUADAS score≥7). The summary estimates of the positive likelihood ratio (PLR), negative likelihood ratio (NLR) and diagnostic odds ratio (DOR) for diagnosing EC were as follows: CEA, 5.94/0.76/9.26; Cyfra21-1, 12.110.59/22.27; p53 antibody, 6.71/0.75/9.60; SCC-Ag, 7.66/0.68/12.41; and VEGF-C, 0.74/0.37/8.12. The estimated summary receiver operating characteristic curves showed that the performance of all five tumor markers was reasonable.

**Conclusions:**

The current evidence suggests that CEA, Cyfra21-1, p53, SCC-Ag and VEGF-C have a potential diagnostic value for esophageal carcinoma.

## Introduction

During the last several decades, the incidence of esophageal squamous cell carcinoma (ESCC) has been declining [1,2]. However, ESCC remains the predominant carcinoma in many countries of east and central Asia [3, 4]. Esophageal cancer (EC), which accounted for 482,300 new cases of cancer in 2008, is the eighth most common cancer worldwide, and has the sixth highest incidence of cancer mortality, with 406,800 deaths registered [5]. Although the prevalence is highest in Africa and Asia, the incidence of adenocarcinoma is rising in western countries and the Americas [6, 7]. Esophageal cancer (EC) is a highly aggressive malignancy due to rapid progression, late diagnosis, and poor prognosis of survival, making the mortality rate of EC patients similar to the rate of the incidence [8, 9]. However, overall survival could be significantly improved by early diagnosis, with a 5-year survival rate of up to 90% [10]. The majority of patients with early EC are asymptomatic and without clinical manifestations. The usual methods of computed tomography (CT) or endoscopic ultrasonography have limited usefulness in early detection because such procedures are often invasive, unpleasant, inconvenient and expensive. In addition, the optimal treatment strategy for advanced EC is still not well established. To our knowledge, there are no suitable diagnostic biomarkers of EC, in contrast to other tumors of the gastrointestinal tract. The spread of malignant tumors is a multistep process involving rapid growth and invasion into the lymph node and blood vessels [11]. Therefore, a low cost, non-invasive, convenient method for routine EC diagnosis is necessary. The detection of biomarkers in serum currently plays an important role in the detection of certain tumors and in monitoring for recurrence or metastasis. Serum tumor markers can be operationally defined as serum molecules whose levels can be used in the diagnosis, prognosis, or clinical management of malignant diseases [12]. Although various biochemical markers have been investigated in the diagnosis and follow-up of EC patients, including p53 antibody, carcinoembryonic antigen (CEA), squamous cell cancer antigen (SCC-Ag), cytokeratin 21–1 fragment (CYFRA21-1), and micro-RNA, there remains a great need to comprehensively and quantitatively summarize the potential diagnostic value of serum biomarkers in esophageal cancer.

## Materials and Methods

### Search strategy and study selection

PubMed, EMBASE, Chinese National Knowledge Infrastructure(CNKI) and Chinese Biomedical Database (CBM) were searched to identify suitable studies up to the 28^th^ of February, 2013; no start data limit was applied. Articles were also identified by use of the related articles function in PubMed and the references of identified articles were searched manually. The search terms were ‘esophageal neoplasm’, ‘blood OR serum’, ‘biomarker OR diagnostic marker’, without language restriction. Conference abstracts and letters to journal editors were excluded because of the limited data contained within.

Two reviewers (Zhang J and Zhu ZL) independently assessed eligible articles based on titles and abstracts, and then the full texts of potentially eligible studies were retrieved for further assessment. Disagreements between the reviewers were resolved by consensus. Studies were included if they met the following inclusion criteria:(1)the performance of biomarkers for the diagnosis of EC were evaluated using a prospective or retrospective design, (2) all cases were diagnosed by a gold standard (pathologic examinations of biopsied specimens), serum must have been collected before any treatment, e.g. chemotherapy or radiotherapy, and controls were without other cancers, and (3) positive values of the cases and controls were reported, and the results of an individual study on diagnostic accuracy could be summarized in a 2×2 table. When the same author reported results obtained from the same patient population in several publications, only the most recent or the most complete report was included in the analysis to avoid overlap between cohorts.

### Assessment of methodological quality

Two dependent reviewers (Zhang J and Liu Y) used 11 items of published QUADAS (quality assessment for studies of diagnostic accuracy) guidelines as a tool to assess the included studies, and disagreements were resolved by consensus. The 11 items were recommended by the Cochrane Collaboration Methods group on screening and diagnostic tests [13]. The items got a “1” score if the item score was “yes”, and aggregate scores totaled 11. Items included covered patient spectrum, reference standard, disease progression bias, verification bias, review bias, clinical review bias, incorporation bias, test execution, study withdrawals, and indeterminate results. The QUADAS tool is presented together with guidelines for scoring each of the items included in the tool.

### Data extraction and management

The final eligible articles were reviewed independently by two reviewers (Zhang J and Zhu ZL), and disagreements were resolved by consensus. The following characteristics studies were extracted: (i) first author, year of publication, country of publication, (ii) participants’ inclusion/exclusion criteria, ethnicity, disease stage, histology stage, diagnostic guidelines, and type of control, (iii) extraction time and storage temperature of the sample, assay method, cut-off value, blindness, and a detailed report of the assay procedure, (iv) the positive value of the cases and controls, and other comparison data (e.g. mean age, sex ratio, smoking, drinking) between cases and controls. If data from any of the above categories were not reported in the primary article, items were treated as “not reported”.

### Statistical analyses

We used standard methods recommended for meta-analysis of diagnostic test evaluations [14]. The positive likelihood ratio (PLR), negative likelihood ratio (NLR) and their 95% confidence interval (CI) were calculated using a random effects model according to the Mantel-Haensed method, and a random effects model based on Der Simonian and Laird [15]. The accuracy measure used was the diagnostic odds ratio (DOR) computed by the Moses’ constant of linear method, which indicates the change in diagnostic performance of the test under study per unit increase in the covariant [16]. Summary receiver operating characteristic curves were used to summarize overall test performance, and the area under the SROC curve (AUC) was calculated. The potential problem associated with sensitivities and specificities of 100% were solved by adding 0.5 to all cells of the diagnostic 2×2 table [14].

We used a chi-squared test to detect statistically significant heterogeneity. Between-study heterogeneity was assessed using I², according to the formula: I^2^ = 100%×(Cochran Q—degrees of freedom)/Cochran Q [17]. To detect cut-off threshold effects, the relationship between sensitivity and specificity was evaluated by using the Spearman correlation coefficient r. In order to check for possible publication bias, a funnel plot of the individual studies was made by plotting logDORs (logarithm of the diagnostic odds ratios) against the sample size [18]. All analyses were undertaken using Meta DiSc statistical software (version 1.4; Ramon y Cajal Hospital, Madrid, Spain) [19] and STATA SE12.0 software (Stata Corporation).

## Results

### Search results and study characteristics

**Fig 1:**
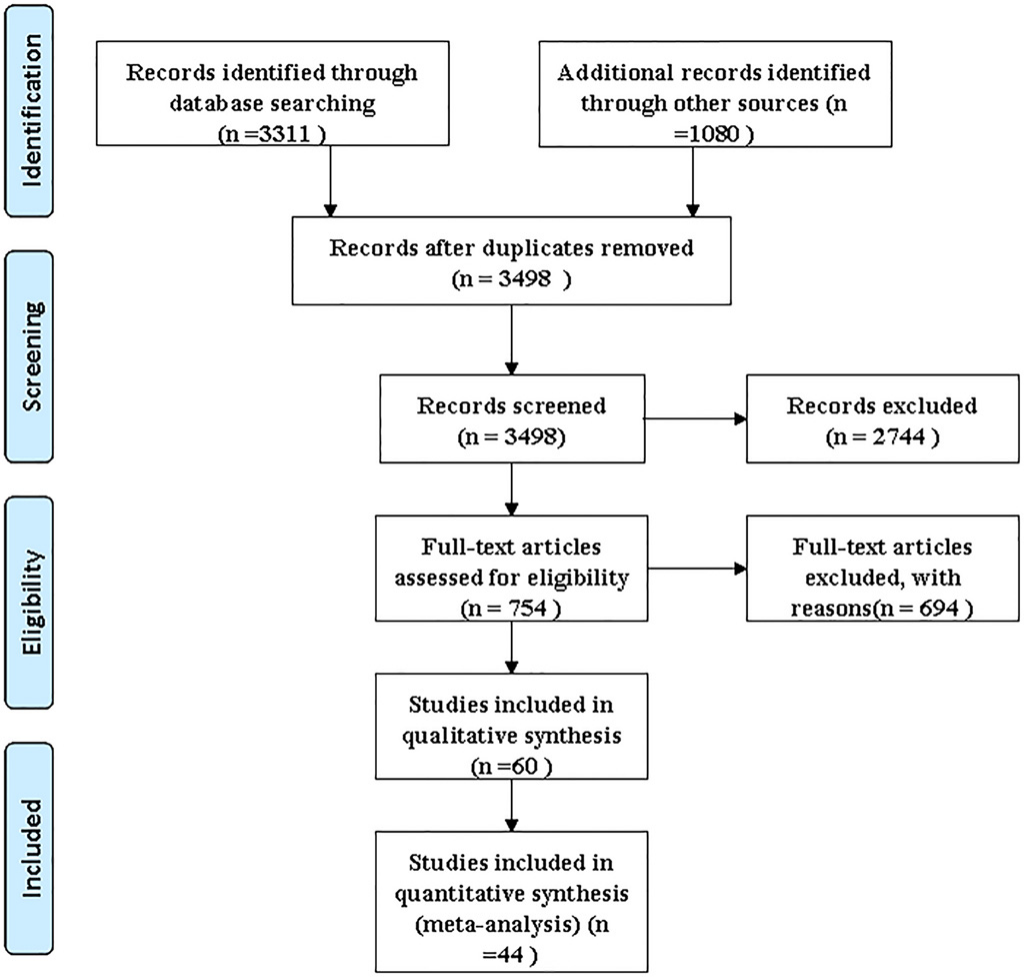
Flow chart of study selection by using electronic database searches.

The study selection is detailed in Fig. 1. Given the overlap between the records identified through database searching and the additional records identified through other sources, 3498 of the 4391 primary studies were strived in abstract form, and the full text was obtained of the 754 full text was obtained of the review. Of these, 379 articles, including a review and case report, were excluded because they provided insufficient information. An additional 315 were excluded because there was no control, and 16 studies with controls were subsequently excluded because they did not allow calculation for sensitivity or specificity. As a result of our database searches and the reference lists of relevant articles, we included in our meta-analysis 44 [12, 20–62] individual studies that comparatively assessed the value of serum biomarkers for EC diagnosis (see Table 1). Computation of the Spearman correlation coefficient between the logit of sensitivity and logit of 1-specificity of CEA, Cyfra21–1, p53 antibody, SCC-Ag and VEGF-C were calculated, indicating no threshold effect [63], and the positive correlation had no statistical significance.

**Table 1:** Main characteristics of the eligible studies sorted by 5 different serum biomarkers.

**First Author**	**Country/Year**	**Ref. Standard**	Assay method*	**Cut-off**	Sen*	**Spec**	Sample collection time*	**Stage I (%)**	**QUADAS**
EUA KLWB MUNCK-WI	Sweden/1988	Unknown	radioimmunoassay techniques	5μg /L	0.39	0.94	unknown	24/95 (25.26%)	3
Matthias Baumann	Germany 1988	Histology	spectrophotometry	5μg /L	0.08	0.57	Before treatment	3/12 (25%)	8
Mortto Uemara	Japan/1990	Unknown	forward sandwich enzyme immunoassay	3 μg/L	0.55	0.91	unknown	unknown	3
Thomas L. Moskal	America/1995	Unknown	RIA	1.5 ng/ml	0.36	1.00	unknown	0	2
Kohtarou Yamamoto	Japan/1997	Histology	ELISA	10 ng/ml	0.04	1.00	Before treatment	3/48 (6.25%)	8
Ren Jun	China/1999	Histology	PCR	unknown	0.17	0.99	unknown	unknown	5
Wang J	China/2000	Histology	ELISA	50 ng/L	0.18	0.92	unknown	unknown	6
Feng XS	China/2000	Histology	HD-2001A biological protein chip	>5 ng/L	0.09	1.00	unknown	unknown	4
Barbara Mroczko	Poland/2007	Histology	a microparticle enzyme immunoassays kit	4.0 ng/ml	0.17	1.00	before operation	0/89 (0%)	7
Ma JY	China/2009	Histology	Multiple tumor markers protein chip kit	5μg /L	0.15	0.85	unknown	unknown	7
Wu XF	China/2009	Histology	ABBOTYEAR2000 Access Immunoassay System	5μg /L	0.70	1.00	unknown	unknown	4
Mao XH	China/2009	Unknown	electrochemilumines cence immunoassay	5 ng/L	0.35	0.87	unknown	unknown	4
Zhao WJ	China/2009	Histology	PCR	5 ng/L	0.06	1.00	unknown	unknown	7
Liu WJ	China/2010	Histology	electrochemilumines cence immunoassay	5 ng/L	0.23	1.00	unknown	unknown	7
Huang ZC	China/2011	Histology	electrochemilumines cence immunoassay electrochemiluminesc ence immunoassay	5 g/L	0.80	0.98	before treatment	24/97 (24.72%)	7
He J	China/2011	Histology	Multiple tumor markers protein chip kit	5 ng/L	0.41	0.97	before operation	unknown	8
Lukaszewicz-Zajzc M.	Poland/2011	Histology	a microparticle enzyme immunoassay kits (MEIA)	4.0 ng/ml	0.30	0.92	before operation	13/53 (24.53%)	7
**Cyfra21-1**									
Kohtarou Yamamoto	Japan/1997	Histology	ELISA	1.5 ng/ml	0.48	1.00	Before treatment	3/48 (6.25%)	8
JENS G.BROCKM ANN	Germany 2000	Histology	immunoradio metric	1.4 ng/ml	0.44	0.94	unknown	unknown	7
Cheng ZZ	China/2008	Histology	electrochemilu minescence immunoassay	3.3 ng/ml	0.37	1.00	Before treatment	unknown	7
Du Xili	China/2010	Histology	ELECSYS	3.3 ng/ml	0.36	1.00	unknown	55/280 (19.64%)	6
Liu WJ	China/2010	Histology	electrochemilu minescence immunoassay	3.3 μg /L	0.40	0.89	unknown	unknown	7
Huang ZC	China/2011	Histology	electrochemilu minescence immunoassay	3.3 μg /L	0.63	0.97	Before treatment	24/97 (24.72%)	7
Dong Y	China/2011	Histology	IMX VIDAS	2.6 ng/ml	0.39	0.98	Before treatment	19/247 (7.69%)	7
**P53**									
Henlen M.	America/1988	Histology	EIA, immunoblot, precipitation	unknown	0.22	0.95	unknown	unknown	8
Parashar K.	India Chandigarh 1988	Unknown	ELISA	unknown	0.30	1.00	unknown	unknown	6
Shimada H.	Japan/2000	Histology	ELISA	Index > = 1.1, Absorption > = 1.6	0.40	1.00	before treatment	unknown	10
Hagiwara N.	Japan/2000	Histology	Sandwich ELISA	unknown	0.28	1.00	before treatment	6/46 (13.0%)	7
Ralhan R.	India New Delhi/2000	Histology	ELISA	unknown	0.60	0.92	before treatment	6/60 (10.0%)	9
Hiroshi SAKAI	Japan/2001	Histology	ELISA	1 u/ml	0.18	0.91	unknown	unknown	7
Kozlowski M.	Poland/2001	Unknown	ELISA	Index > = 1.1	0.27	1.00	before diagnosis	4/75 (5.3%)	7
Shimada H.	Japan/2002	Histology	ELISA	1.3 U/ml	0.27	0.95	unknown	50/105 (47.6%)	8
Shimada H.	Japan/2003	Unknown	ELISA	1.3 U/ml	0.30	0.94	unknown	unknown	6
Wang M.H.	China/2004	Histology	ELISA	Index > = 1.1, Absorption > = 1.6	0.47	1.00	before treatment	10/38 (26.3%)	9
Hiroyuki K.	Japan/2005	Histology	ELISA	1.3 U/ml	0.32	1.00	unknown	13/57 (22.8%)	8
Megliorino R.	China/2005	Histology	ELISA	Normal mean+3SD	0.14	0.98	before chemotherapy	unknown	8
Looi K.	China/2006	Unknown	ELISA	Normal mean+3SD	0.07	0.99	before diagnosis	unknown	7
Muller M.	Germany/2006	Histology	immunoblot	unknown	0.20	1.00	unknown	unknown	7
Cai H.Y.	China/2008	Histology	ELISA	unknown	0.39	1.00	before chemo therapy	10/46 (21.7%)	8
Wu M.	China/2010	Unknown	ELISA	unknown	0.14	0.99	unknown	unknown	6
**SCC-Ag**									
Kohtarou Yamamoto	Japan/1997	Histology	ELISA	1.5 ng/ml	0.25	1.00	Before treatment	3/48 (6.25%)	8
Hagiwara N.	Japan/2000	Histology	ELISA	1.5 ng/ml	0.13	0.92	before treatment	6/46 (13.0%)	7
Barbara Mroczko	Poland/2007	Histology	chemiluminescenc assays (CMIA)	2 ng/ml	0.64	0.93	before operation	0/89 (0%)	7
Cheng ZZ	China/2008	Histology	ELISA	2 ng/ml	0.26	1.00	Before treatment	unknown	7
Cao Mei	China/2009	Histology	ELISA	1.2 ng/ml	0.41	1.00	before operation	11/108 (10.19%)	6
Mao XH	China/2009	Unknown	MEIA	1.5 μg/L	0.39	0.92	unknown	unknown	4
Huang ZC	China/2011	Histology	MEIA	1.5 μg /L	0.43	0.91	Before treatment	24/97 (24.72%)	7
Dong Y	China/2011	Histology	IMX VIDAS	1.5 ng/ml	0.23	0.98	Before treatment	19/247 (7.69%)	7
Lukaszewicz Zajzc M.	Poland/2011	Histology	ELISA	2 ng/ml	0.25	0.96	before operation	13/53 (24.53%)	7
Atsuki Lkeda	Japan/2011	Histology	chromatography	unknown	0.47	0.92	unknown	1/15 (6.67%)	6
M. Cao	China/2011	Histology	ELISA	1.2 ng/ml	0.38	1.00	unknown	15/56 (26.79)	7
**VEGF-C**									
M. Krzystek-Korpacka	Poland/2011	Histology	unknown	12.14 μg/ml	0.85	0.53	preceding treatment	unknown	7
Miroslaw Kozlowski	Poland/2010	Histology	ELISA	unknown	0.64	0.80	before surgery	11/149 (7.38%)	7
Malgorzata Krzystek-Korpacka	Poland/2007	Histology	immuno enzymatic	13.14 ng/ml	0.79	0.76	at admission	0	7
Malgorzata krz-Kor	Poland/2006	Histology	ELISA	14.57 ng/ml	0.70	0.81	unknown	0	7

Note: ELISA* = Enzyme-linked immunosorbent assay; TP* = true positives, FP* = false positives, FN* = false negatives, TN* = true negatives; Abs* = Antibody.

Assay method*：ELISA or ELISA+western = 1; other = 0; unknown = 2.

Formula 1*: normal+2SD

Formula 2*: P53 control protein ratio >1.3

Formula 3*: density>1030

Formula 4*: optical density>0.11

Formula 5*: manufacturer’s formula

Formula 6*: the differences in the absorption values between the positive walls and controls was greater than 0.5

Formula 7*: differences higher than 0.15 were scored positive

Formula 8*: index≥1.1,Absorption> = 1.6

Formula 9*: ≥2.3-times of control

Formula 10*: mean+3SD

Note: Sample collection time*: before diagnosis = 1; before treatment = 0; unknown = 2.

### CEA

**Fig 2:**
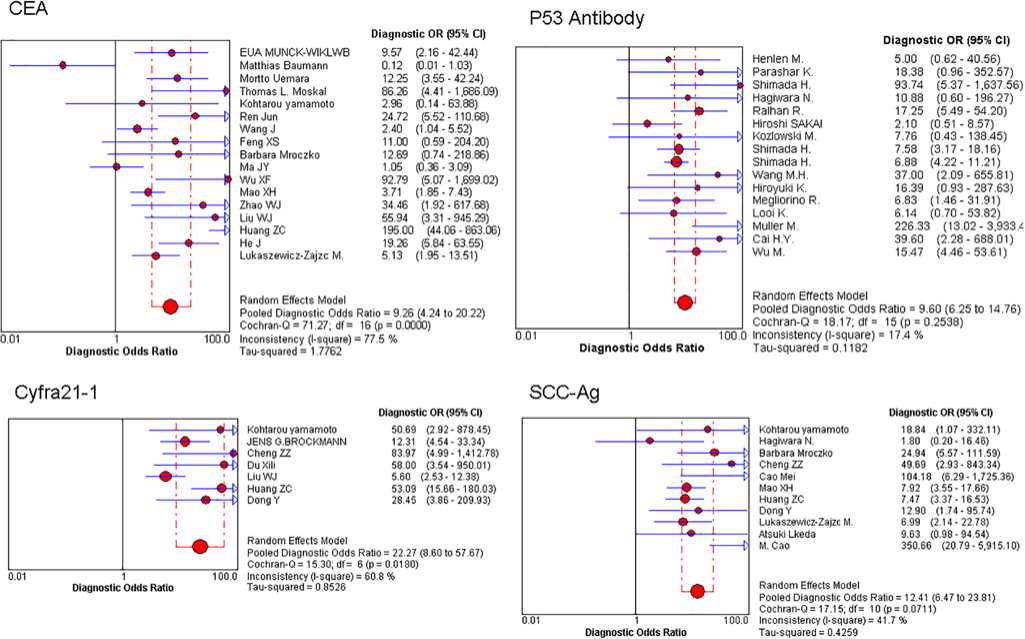
Forest plot of estimates of the diagnostic odds ratio (DOR) for CEA, Cyfra21–1, p53, and SCC-Ag in the diagnosis of EC. Point estimates of the diagnostic odds ratio from each study are shown as solid circles. Error bars are 95% confidence intervals.

Seventeen studies (cases = 1017, controls = 2877) met the inclusion criteria for the meta-analysis. Approximately 52.94% (9/17) of the included studies were of high quality (QUADAS score≥7). The sensitivity and specificity of the 17 selected CEA studies [12, 36–51] ranged from 8% to 70%, and from 57% to 100%, respectively; the pooled estimates and the corresponding PLR, NLR, DOR and AUC are shown in Table 2. A pooled PLR of 5.94 (95% CI: 3.24–10.89) suggests that patients with EC have a nearly 6-fold higher chance of being CEA test-positive compared with patients without EC. Also, the pooled negative likelihood ratio was 0.76 (95% CI: 0.67–0.86). For all 17 studies, the pooled DOR was 9.26 (95% CI:4.24–20.22). There was heterogeneity between studies (Fig. 2). The symmetrical SROC for CEA gave an AUC of 0.74 (Fig. 2). In our study, the AUC for sp53 antibody was 0.71. Thus CEA had reasonable accuracy in terms of differential diagnosis in cases of EC.

**Table 2:** Diagnostic accuracy of CEA, Cyfra21–1, p53, SCC-Ag, and VEGF-C for EC.

	No.	Case	Control	PLR	NLR	DOR	AUC
		(n)	(n)	(95%CI) ^*^	(95%CI) ^*^	(95%CI) ^*^	
CEA	17	1017	2877	5.94 (3.24–10.89)	0.76 (0.67–0.86)	9.26 (4.24–20.22)	0.71
Cyfra21–1	7	872	483	12.11(5.02–29.24)	0.59 (0.52–0.66)	22.27 (8.60–57.67)	0.58
P53	16	1096	2384	6.71(4.61–9.75)	0.75(0.69–0.82)	9.60 (6.25–14.76)	0.73
SCC-Ag	11	918	867	7.66(4.24–13.83)	0.68 (0.61–0.77)	12.41 (6.47–23.81)	0.69
VEGF-C	4	363	195	2.74 (1.85–4.07)	0.37 (0.29–0.47)	8.12 (5.37–12.27)	0.81

Note:PLR: positive likelihood ratio, NLR: negative likelihood ratio, DOR: diagnostic odds ratio, AUC: the area under the SROC curve; PLR (95% CI)^*^, DOR (95% CI)^*^ and NLR (95% CI)^*^ were calculated using a random effect model.

### Cyfra21–1

Seven studies (cases = 1017, controls = 2877) met the inclusion criteria for the meta-analysis. Approximately 85.71% (6/7) of the included studies were of high quality (QUADAS score≥7). The sensitivity and specificity of the 7 selected Cyfra21–1 studies [39, 48, 50, 52, 55, 57, 58] ranged from 36% to 63%, and from 89% to 100%, respectively; the pooled estimates and the corresponding PLR, NLR, DOR and AUC are shown in Table 2. A pooled PLR of 12.11(95% CI: 5.02–29.24) suggests that patients with EC have a nearly 12-fold higher chance of being Cyfra21–1 test-positive compared with patients without EC. Also, the pooled negative likelihood ratio was 0.59 (95% CI: 0.52–0.66). For all 7 studies, the pooled DOR was 22.27 (95% CI: 8.20–57.67) (Fig. 2). There was heterogeneity between studies. The symmetrical SROC of Cyfra21–1 had an AUC of 0.58 (Fig. 2).

### P53 antibody

Sixteen studies [20–35] (cases = 1079, controls = 2260) met the inclusion criteria for the meta-analysis. Approximately 53.33% (8/15) of the included studies were of high quality (QUADAS score≥8). The sensitivity and specificity of the 16 selected studies [20–35] ranged from 14% to 60%, and from 91% to 100%, respectively; the pooled estimates and the corresponding PLR and NLR are shown in Table 2. A pooled PLR of 6.71 (95% CI: 4.61–9.75) suggests that patients with EC have a nearly 7-fold higher chance of being s-p53-antibody test-positive compared with patients without EC. Also, the pooled negative likelihood ratio was 0.75 (95% CI: 0.69–0.82). For all 16 studies, the pooled DOR was 9.60 (95%CI: 6.25–14.76) (Fig. 2). There was heterogeneity between studies. Fig. 2 shows the symmetrical SROC for s-p53 antibody (serum p53 antibody) has an AUC of 0.73.

### SCC-Ag

Eleven studies [22, 39, 43, 45, 50–56] (cases = 918, controls = 867) met the inclusion criteria for the meta-analysis. Approximately 72.73% (8/11) of the included studies were of high quality (QUADAS score≥8). The sensitivity and specificity of the 11 selected studies ranged from 13% to 64%, and from 91% to 100%, respectively; the pooled estimates and the corresponding PLR and NLR are shown in Table 2. A pooled PLR of 7.66 (95% CI: 4.24–13.83) suggests that patients with EC have a nearly 8-fold higher chance of being SCC-Ag test-positive compared with patients without EC. Also, the pooled negative likelihood ratio was 0.68 (95% CI: 0.61–0.77). For all 16 studies, the pooled DOR was 12.41 (95%CI: 6.47–23.81) (Fig. 2). There was heterogeneity between studies. The symmetrical SROC of s-p53 antibody gives an AUC of 0.69.

### VEGF-C

**Fig 3:**
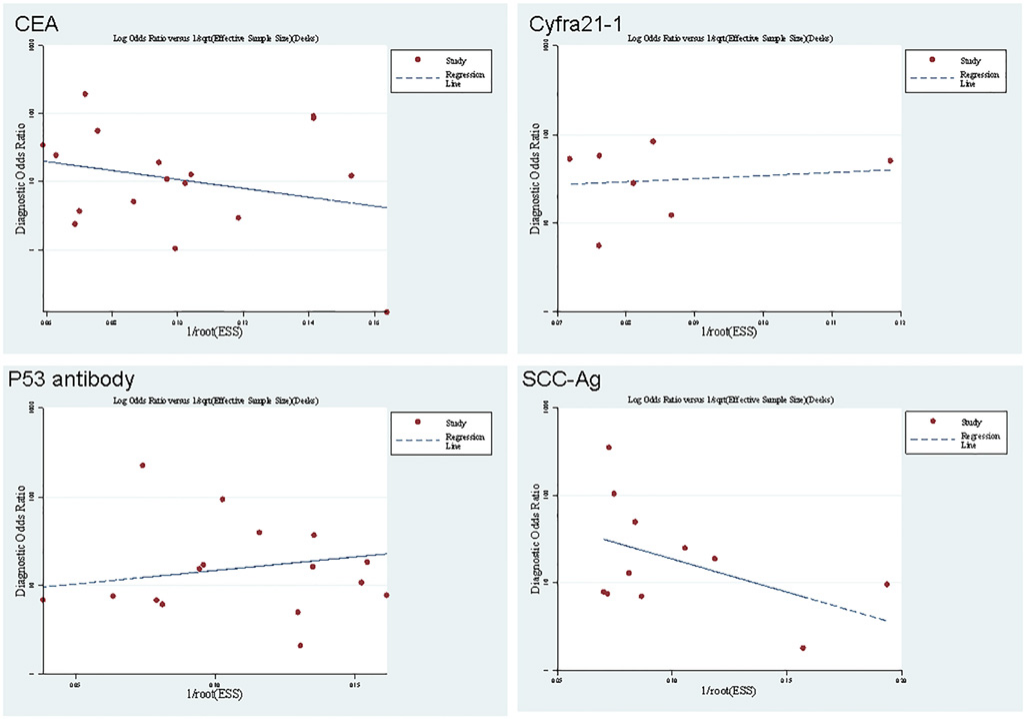
Funnel plot for the assessment of potential bias in CEA, Cyfra21–1, p53 and SCC-Ag assays. The funnel graph plots the DOR (diagnostic odds ratio) against the 1/root (effective sample size), the dotted line is the regression line.

Four studies (cases = 363, controls = 195) met the inclusion criteria for the meta-analysis. All four included studies were of high quality (QUADAS score≥7), with sensitivity and specificity ranging from 64% to 85% and from 53% to 81%, respectively; The pooled estimates and the corresponding PLR and NLR are shown in Table 2. A pooled PLR of 2.74 (95% CI: 1.85–4.07) suggests that patients with EC have a nearly 3-fold higher chance of being VEGF-C test-positive compared with patients without EC. Also, the pooled negative likelihood ratio was 0.37 (95% CI: 0.29–0.47). For all four studies, the pooled DOR was 8.12 (95% CI: 5.37–12.27). There was heterogeneity between studies. Fig. 3 shows the symmetrical SROC of VEGF-C has an AUC of 0.81.

### Publication bias

Publication bias is assessed visually by using a scatter plot of the inverse square root of the effective sample size (1/ESS^1/2^) versus the diagnostic log odds ratio (lnDOR), which should have a symmetrical funnel shape when publication bias is absent [64]. Formal testing for publication bias can be conducted by a regression of lnDOR against 1/ESS^1/2^, weighting by ESS [64], with a slope coefficient of P < 0.05 indicating significant asymmetry. Although meta-analysis itself has some bias, the results showed no publication bias in this meta-analysis (CEA, p = 0.339; Cyfra21–1, p = 0.841; p53, p = 0.408; SCC-Ag, p = 0.397). The funnel plots (Fig. 3) for publication bias also showed symmetry.

## Discussion

Making a differential diagnosis between EC and non-EC is a critical clinical problem and conventional tests are not always. Usually, histological examination is used to diagnose EC. More and more studies have been focused on the detection of serological tumor markers in EC to evaluate the diagnostic and clinical usefulness. The overall specificity of CEA, Cyfra21–1, p53 antibody, SCC-Ag and VEGF-C were 98.0%, 97.8%, 98.4%, 98.0% and 73.2%, respectively. The summary estimate of the sensitivities for the five tumor markers were, however, all quite low and were more variable than the specificity. These data suggest a potential role for determination of these tumor markers in confirming EC. However, these tests maximize specificity at the cost of sensitivity, and this trade-off has significant clinical implications. By contrast with the higher specificity, these tumor markers had low sensitivities that were not sufficiently low to exclude non-EC when the tumor marker concentrations are lower than the cut-off values. Negative tests do not therefore mean absence of EC, and patients with negative tumor marker results have a fairly high chance of having EC.

The SROC curve presents a global summary of test performance and shows the trade-off between sensitivity and specificity. On the other hand, mean AUC ranged from 0.73 to 0.88, suggesting that the overall accuracy of tumor markers in diagnosing EC is not as high as expected. The DOR is a single indicator of test accuracy that combines the data from sensitivity and specificity into a single number. The DOR of a test is the ratio of the odds of a positive test result in a subject with the disease relative to the odds of a positive test result in a subject without the disease [65]. The value of a DOR ranges from 0 to infinity, with higher values indicating better discriminatory test performance (higher accuracy). A DOR of 1.0 indicates that a test does not discriminate between patients with the disorder and those without it. A DOR value of 1.00 suggests improper test interpretation (a greater proportion of negative test results in the group with disease) [65]. In the present meta-analysis, we find that the mean DOR values for CEA, Cyfra21–1, p53 antibody, SCC-Ag and VEGF-C were 16.67, 34.56, 22.88, 24.99 and 7.97, respectively, indicating that, although not as good as expected, measurement of these four tumor markers could be helpful in the diagnosis of EC. Since the SROC curve and the DOR are not easy to interpret and use in clinical practice, and since the likelihood ratios are considered more clinically meaningful [66, 67], we also present both the PLR and NLR as measures of diagnostic accuracy for the tumor markers. Likelihood ratios of 10 or 0.1 generate large and often conclusive shifts from pre-test to post-test probability (indicating high accuracy) [67]. Out data show that overall PLR values for CEA, Cyfra21–1, p53 antibody, SCC-Ag and VEGF-C ranged from 7.97 to 34.55, suggesting that patients with EC have a nearly 8- to 35-fold higher chance of being positive compared to patients without EC. On the other hand, the mean NLR values of CEA, Cyfra21–1, p53 antibody, SCC-Ag and VEGF-C ranged from 0.35 to 0.76, so if the assay results are negative, the probability that this patient has EC ranges from 35% to 76%, which is too high to rule out EC. In addition to the five tumor markers analyzed in the present meta-analysis, other biomarkers such as plasma deoxyribonucleic acid, serum Dickkopf-1, matrix metalloproteinase 9, matrix metalloproteinase 7, serum interleukin 6, serum makorin 1 antibody, human leukocyte antigen-G, serum TRIM21 antibody, serum hyaluronic acid, cell division cycle 25B antibody, heat shock protein 70 antibody, glutathione S-transferase n, have been evaluated for their use in the diagnosis of EC. However, there were no sufficient eligible primary studies for our meta-analysis.

Although we tried to avoid bias in the process of identifying studies, screening, assessing, data extraction, and data analyses, the present study has several limitations. First, the exclusion of conference abstracts and letters to journal editors may have led to publication bias, an inflation of accuracy estimates due to preferential acceptance of papers reporting favorable results, and the potential for publication bias in studies included in the present meta-analysis. Second, we did not calculate the diagnostic accuracy for early stage (stage I-II) cancers because sufficient raw data was not provided. Although we aimed to evaluate the diagnostic value of tumor markers for the early diagnosis of the cancer, cancer patients regardless of disease stage were used to evaluate the diagnostic power because of the limited amount of information. Primary data was unavailable for investigation of elevated or decreased tumor marker-positive values as a function of tumor type, histology, age, or degree. Also, because of lack of required data reported in the original publications, we did not calculate the diagnostic value of the combination of tumor markers. Thirdly, we excluded 20 studies because they did not provide data allowing construction of 2×2 tables. We did not contact authors to obtain further data, potentially resulting in biased results and less precise estimates of pooled diagnostic accuracy. Finally, we only included five biomarkers because the other 12 biomarkers could not be pooled as lacking of insufficient studies. As we all known, meta-analysis must pool two studies at least. The last but not the least, in all 44 studies, cancer patients diagnosed by histology was regarded as positive. However, the negative controls without cancer that were healthy or had benign disease were not diagnosed by histology. In addition, most of the studies did not report whether the investigators were blinded. Therefore, such non-strict designs could exaggerate the diagnostic accuracy and lead to bias due to unfavorable representation of the participants.

The accuracy of tumor marker determinations for EC seems to be similar to that of conventional tests such as cytological examination, which has high specificity and low sensitivity. This similarity might make tumor markers less useful in practice because they do not have test properties that complement the properties of conventional tests. However, it should be pointed out that, to date, there are insufficient related studies to evaluate the diagnostic accuracy of the combination of two or more tumor markers in EC.

In conclusion, current evidence suggests that CEA, Cyfra21–1, p53 antibody, SCC-Ag and VEGF-C are highly specific, but insufficiently sensitive to diagnose EC. Patients with cancer have a higher chance of being CEA-, Cyfra21–1-, p53 antibody-, SCC-Ag- and VEGF-C-positive compared to patients without cancer. Although CEA, Cyfra21–1, p53, SCC-Ag and VEGF-C have a potential diagnostic value for esophageal carcinoma., we do not recommend using one tumor marker alone for the diagnosis of EC. Further studies may need to identify patterns of multiple biomarkers to further increase the power of EC detection.

## Supporting Information

S1 PRISMA Checklist (DOC)
