# Supplementary material for: Diagnostic Value of Multiple Tumor Markers for Patients with Esophageal Carcinoma
Source: PLoS One. 2015 Feb 18;10(2):e0116951. doi: 10.1371/journal.pone.0116951 (PMC4333286; doi:10.1371/journal.pone.0116951)
Supplement: S1 PRISMA Checklist — (DOC) [file pone.0116951.s001.doc]

| **Section/topic** | **#** | **Checklist item** | **Reported on page #** |
| --- | --- | --- | --- |
| **TITLE** | | |  |
| Title | 1 | Diagnostic value of multiple tumor markers for patients with esophageal carcinoma | #1 |
| **ABSTRACT** | | |  |
| Structured summary | 2 | Background: Various studies assessing the diagnostic value of serum tumor markers in patients with esophageal cancer remain controversial. This study aims to comprehensively and quantitatively summarize the potential diagnostic value of 5 serum tumour markers in esophageal cancer.Methods: We systematically searched PubMed, Embase, Chinese National Knowledge Infrastructure (CNKI) and Chinese Biomedical Database (CBM), through February 28, 2013, without language restriction. Studies were assessed for quality using QUADAS (quality assessment of studies of diagnostic accuracy). The positive likelihood ratio (PLR) and negative likelihood ratio (NLR) were pooled separately and compared with overall accuracy measures using diagnostic odds ratios (DORs) and symmetric summary receiver operating characteristic (SROC) curves. Results: Of 4391 studies initially identified, 44 eligible studies including five tumor markers met the inclusion criteria for the meta-analysis, while meta-analysis could not be conducted for 12 other tumor markers. Approximately 79.55% (35/44) of the included studies were of relatively high quality (QUADAS score≥7). The summary estimates of the positive likelihood ratio (PLR), negative likelihood ratio (NLR) and diagnostic odds ratio (DOR) for diagnosing EC were as follows: CEA, 5.94/0.76/9.26; Cyfra21-1, 12.110.59/22.27; p53 antibody, 6.71/0.75/9.60; SCC-Ag, 7.66/0.68/12.41; and VEGF-C, 0.74/0.37/8.12. The estimated summary receiver operating characteristic curves showed that the performance of all five tumor markers was reasonable.  Conclusions: The current evidence suggests that CEA, Cyfra21-1, p53, SCC-Ag and VEGF-C may serve as a potential useful markers for routine screening in asymptomatic high-risk patient groups. | #2 |
| INTRODUCTION | | |  |
| Rationale | 3 | Esophageal cancer (EC) is a highly aggressive malignancy due to rapid progression, late diagnosis, and poor prognosis of survival, making the mortality rate of EC patients similar to the rate of the incidence . However, overall survival could be significantly improved by early diagnosis, with a 5-year survival rate of up to 90% . The majority of patients with early EC are asymptomatic and without clinical manifestations. The usual methods of computed tomography (CT) or endoscopic ultrasonography have limited usefulness in early detection because such procedures are often invasive, unpleasant, inconvenient and expensive. In addition, the optimal treatment strategy for advanced EC is still not well established. To our knowledge, there are no suitable diagnostic biomarkers of EC, in contrast to other tumors of the gastrointestinal tract. The spread of malignant tumors is a multistep process involving rapid growth and invasion into the lymph node and blood vessels . Therefore, a low cost, non-invasive, convenient method for routine EC diagnosis is necessary. The detection of biomarkers in serum currently plays an important role in the detection of certain tumors and in monitoring for recurrence or metastasis. Serum tumor markers can be operationally defined as serum molecules whose levels can be used in the diagnosis, prognosis, or clinical management of malignant diseases . | #3, #4 |
| Objectives | 4 | This study aims to comprehensively and quantitatively summarize the potential diagnostic value of 5 serum tumour markers in esophageal cancer. | #3, #4 |
| METHODS | | |  |
| Protocol and registration | 5 | No protocol and registration . |  |
| Eligibility criteria | 6 | Studies were included if they met the following inclusion criteria:⑴the performance of biomarkers for the diagnosis of EC were evaluated using a prospective or retrospective design, ⑵ all cases were diagnosed by a gold standard (pathologic examinations of biopsied specimens), serum must have been collected before any treatment, e.g. chemotherapy or radiotherapy, and controls were without other cancers, and ⑶ positive values of the cases and controls were reported, and the results of an individual study on diagnostic accuracy could be summarized in a 2×2 table. When the same author reported results obtained from the same patient population in several publications, only the most recent or the most complete report was included in the analysis to avoid overlap between cohorts. | #5, #6 |
| Information sources | 7 | PubMed, EMBASE, Chinese National Knowledge Infrastructure(CNKI) and Chinese Biomedical Database (CBM) were searched to identify suitable studies up to the 28th of February, 2013; no start data limit was applied. | #5 |
| Search | 8 | #1 esophageal neoplasms[all fields]  #2 esophageal or esophagus or oesophagus or oesophageal[all fields]  #3 cancer or carcinoma or adenocarcinoma malignan or tumor or tumour or neoplasm[all fields]  #4 #2 and #3  #5 ESCC OR EAC OR OSCC OR OAC [all fields]  #6 #1 or #4 or #5  #7 blood OR serum OR serological OR seropositive OR seropositivity OR serum antibody OR sera OR plasma[all fields]  #8 "Biological Markers"[Mesh]  #9 diagnostic marker [all fields]  #10 non-invasive predictor [ all fields]  #11 #8 OR #9 OR #10  #12 #6 AND #7 AND #11 limits: human | #5 |
| Study selection | 9 | Two reviewers (Zhang J and Zhu ZL) independently assessed eligible articles based on titles and abstracts, and then the full texts of potentially eligible studies were retrieved for further assessment. Disagreements between the reviewers were resolved by consensus. | #5 |
| Data collection process | 10 | The following characteristics studies were extracted: (i) first author, year of publication, country of publication, (ii) participants’ inclusion/exclusion criteria, ethnicity, disease stage, histology stage, diagnostic guidelines, and type of control, (iii) extraction time and storage temperature of the sample, assay method, cut-off value, blindness, and a detailed report of the assay procedure, (iv) the positive value of the cases and controls, and other comparison data (e.g. mean age, sex ratio, smoking, drinking) between cases and controls. If data from any of the above categories were not reported in the primary article, items were treated as "not reported." | #6，#7 |
| Data items | 11 | outcome: the positive value of the cases and controls, and other comparison data (e.g. mean age, sex ratio, smoking, drinking) between cases and controls | #7 |
| Risk of bias in individual studies | 12 | In all 44 studies, cancer patients diagnosed by histology were regarded as positive. However, the negative controls without cancer who were healthy or had benign disease were not diagnosed by histology. In addition, most of the studies did not report whether the investigators were blinded. Therefore, such non-strict designs could exaggerate the diagnostic accuracy and lead to bias due to unfavorable representation of the participants. | #13 |
| Summary measures | 13 | The positive likelihood ratio (PLR), negative likelihood ratio (NLR) and their 95% confidence interval (CI) were calculated using a random effects model according to the Mantel-Haensed method, and a random effects model based on Der Simonian and Laird . The accuracy measure used was the diagnostic odds ratio (DOR) computed by the Moses’ constant of linear method, which indicates the change in diagnostic performance of the test under study per unit increase in the covariant . Summary receiver operating characteristic curves were used to summarize overall test performance, and the area under the SROC curve (AUC) was calculated. The potential problem associated with sensitivities and specificities of 100% were solved by adding 0.5 to all cells of the diagnostic 2×2 table . We used a chi-squared test to detect statistically significant heterogeneity. Between-study heterogeneity was assessed using I², according to the formula: I² = 100%×(Cochran Q –degrees of freedom)/Cochran Q . To detect cut-off threshold effects, the relationship between sensitivity and specificity was evaluated by using the Spearman correlation coefficient r. In order to check for possible publication bias, a funnel plot of the individual studies was made by plotting logDORs (logarithm of the diagnostic odds ratios) against the sample size . | #7, #8 |
| Synthesis of results | 14 | The summary estimates of the positive likelihood ratio (PLR), negative likelihood ratio (NLR) and diagnostic odds ratio (DOR) for diagnosing EC were as follows: CEA, 5.94/0.76/9.26; Cyfra21-1, 12.110.59/22.27; p53 antibody, 6.71/0.75/9.60; SCC-Ag, 7.66/0.68/12.41; and VEGF-C, 0.74/0.37/8.12. The estimated summary receiver operating characteristic curves showed that the performance of all five tumor markers was reasonable. | #8,#9 |

Page 1 of 2

| **Section/topic** | **#** | **Checklist item** | **Reported on page #** |
| --- | --- | --- | --- |
| Risk of bias across studies | 15 | Quality assessment based on QUADAS guidelines was conducted on all 44 studies included for systematic review.. | #6 |
| Additional analyses | 16 | To detect cut-off threshold effects, the relationship between sensitivity and specificity was evaluated by using the Spearman correlation coefficient r. In order to check for possible publication bias, a funnel plot of the individual studies was made by plotting logDORs (logarithm of the diagnostic odds ratios) against the sample size | #7 |
| RESULTS | | |  |
| Study selection | 17 | Fig.1 Flow chart of study selection by using electronic database and other sources. | #8 |
| Study characteristics | 18 | Table 1. Main characteristics of the 44 eligible studies | #11 |
| Risk of bias within studies | 19 | Publication bias is assessed visually by using a scatter plot of the inverse of the square root of the effective sample size (1/ESS1/2) versus the diagnostic log odds ratio (lnDOR) ,the results showed no publication bias in this meta-analysis (p=0.000) . | #10,#11 |
| Results of individual studies | 20 | Table 1. Main characteristics of the eligible studies sorted by 5 different serum biomarkers. | #18 |
| Synthesis of results | 21 | Table 1. Main characteristics of the eligible studies sorted by 5 different serum biomarkers.  Table 2. Diagnostic accuracy of CEA, Cyfra21-1, p53, and SCC-Ag for EC.  Figure 1. Flow chart of study selection by using electronic database searches.  Figure 2. Forest plot of estimates of the diagnostic odds ratio (DOR) for CEA, Cyfra21-1, p53, and SCC-Ag in the diagnosis of EC. Point estimates of the diagnostic odds ratio from each study are shown as solid circles. Error bars are 95% confidence intervals. | #18, #20 |
| Risk of bias across studies | 22 | Figure 3 Funnel plot for the assessment of potential bias in CEA, Cyfra21-1, p53 and SCC-Ag assays. The funnel graph plots the DOR (diagnostic odds ratio) against the 1/root (effective sample size), the dotted line is the regression line. | #20 |
| Additional analysis | 23 | The synthesis of results(sensitive analysis) were not shown. |  |
| DISCUSSION | | |  |
| Summary of evidence | 24 | The overall specificity of CEA, Cyfra21-1, p53 antibody，SCC-Ag and VEGF-C were 98.0%, 97.8%, 98.4%, 98.0% and 73.2%, respectively. The summary estimate of the sensitivities for the five tumor markers were, however, all quite low and were more variable than the specificity. These data suggest a potential role for determination of these tumor markers in confirming EC. However, these tests maximize specificity at the cost of sensitivity, and this trade-off has significant clinical implications. By contrast with the higher specificity, these tumor markers had low sensitivities that were not sufficiently low to exclude non-EC when the tumor marker concentrations are lower than the cut-off values. Negative tests do not therefore mean absence of EC, and patients with negative tumor marker results have a fairly high chance of having EC. | #11 |
| Limitations | 25 | First, the exclusion of conference abstracts and letters to journal editors may have led to publication bias, an inflation of accuracy estimates due to preferential acceptance of papers reporting favorable results, and the potential for publication bias in studies included in the present meta-analysis. Second, we did not calculate the diagnostic accuracy for early stage (stage I-II) cancers because sufficient raw data was not provided. Although we aimed to evaluate the diagnostic value of tumor markers for the early diagnosis of the cancer, cancer patients regardless of disease stage were used to evaluate the diagnostic power because of the limited amount of information. Primary data was unavailable for investigation of elevated or decreased tumor marker-positive values as a function of tumor type, histology, age, or degree. Also, because of lack of required data reported in the original publications, we did not calculate the diagnostic value of the combination of tumor markers. Thirdly, we excluded 20 studies because they did not provide data allowing construction of 2×2 tables. We did not contact authors to obtain further data, potentially resulting in biased results and less precise estimates of pooled diagnostic accuracy. Finally, we only included five biomarkers because the other 12 biomarkers could not be pooled as lacking of insufficient studys. As we all known, meta-analysis must pool two studys at least. | #12,#13 |
| Conclusions | 26 | In conclusion, current evidence suggests that CEA, Cyfra21-1, p53 antibody，SCC-Ag and VEGF-C are highly specific, but insufficiently sensitive to diagnose EC. Patients with cancer have a higher chance of being CEA-, Cyfra21-1-, p53 antibody-，SCC-Ag- and VEGF-C-positive compared to patients without cancer. Although CEA, Cyfra21-1, p53, SCC-Ag and VEGF-C may serve as useful markers for routine screening in asymptomatic high-risk patient groups, we do not recommend using one tumor marker alone for the diagnosis of EC. Further studies may need to identify patterns of multiple biomarkers to further increase the power of EC detection. | #13 |
| **FUNDING** | | |  |
| Funding | 27 | No any funding and support . |  |

*From:*  Moher D, Liberati A, Tetzlaff J, Altman DG, The PRISMA Group (2009). Preferred Reporting Items for Systematic Reviews and Meta-Analyses: The PRISMA Statement. PLoS Med 6(6): e1000097. doi:10.1371/journal.pmed1000097

For more information, visit: **www.prisma-statement.org**.

Page 2 of 2
